# Fabrication and characterization of tretinoin-loaded nanofiber for topical skin delivery

**DOI:** 10.1186/s40824-020-00186-3

**Published:** 2020-03-02

**Authors:** Saba Khoshbakht, Farzin Asghari-Sana, Anahita Fathi-Azarbayjani, Yaeghob Sharifi

**Affiliations:** 1 Student Research Committee, Urmia University of Medical Science, Urmia, Iran; 2 Solid Tumor Research Center, Urmia University of Medical Sciences, Urmia, Iran; 3 Department of Pharmaceutics, School of Pharmacy, Urmia University of Medical Sciences, Urmia, Iran; 4 Department of Clinical Microbiology, School of Medicine, Urmia University of Medical Sciences, Urmia, Iran

**Keywords:** Tretinoin, Erythromycin, Nanofiber, Stability, Drug release

## Abstract

**Background:**

Tretinoin or all-trans retinoic acid is used in the treatment of acne vulgaris and photo-aging. This work aims to develop tretinoin-loaded nanofibers as a potential anti-acne patch and to investigate its physicochemical characteristics.

**Method:**

Nanofibers were produced via electrospinning method and surface topography was evaluated by Field Emission Scanning Electron Microscopy (FESEM). The functional groups of polymer and the drug molecule and the possible interactions were studied by Fourier Transform Infrared Spectroscopy (FTIR). Drug release studies were carried out by total immersion method at 25 °C and 32 °C. Tretinoin stability was evaluated at room temperature and fridge for 45 days. The possibility of synergistic antibacterial activity of tretinoin and erythromycin combination was investigated on *Staphylococcus aureus* (ATCC® 25923™) and (ATCC® 29213™) by Kirby Bauer disc diffusion method.

**Results:**

Uniform fibers without drug crystals were fabricated via electrospinning. Drug-loaded nanofibers show inherent stability under various storage conditions. Electrospun nanofibers showed a prolonged release of tretinoin. The stability of formulations in FT was higher than RT. Disc diffusion tests did not show any synergism in the antibacterial activity of erythromycin when used in combination with tretinoin.

**Conclusion:**

It can be anticipated that the easy fabrication, low costs and dosing frequency of the construct reported here provide a platform that can be adapted for on-demand delivery of tretinoin.

**Graphical abstract:**

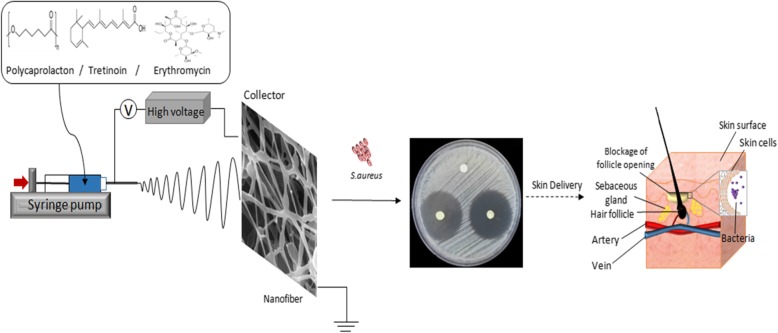

## Introduction

Electrospinning is widely employed for the fabrication of high-quality nanofiber at low production cost. Nanofibers wound dressings can mimic extracellular matrix and minimize scar formation. Other application that can benefit from the favorable properties of nanofibers includes separation of contaminants from water and molecule filtration offered by the high efficiency and low resistance structure. Various hydrophilic and hydrophobic drug molecules can be incorporated in nanofibers. The selection of a specific polymer with a known molecular weight can help in the design of a rapid, sustained or pulsatile drug delivery system [1, 2].

Polycaprolactone (PCL) is a biodegradable and biocompatible synthetic hydrophobic polymer with a semi-crystalline structure. It has been approved by the US Food and Drug Administration (FDA) and is being widely used in the design of various drug delivery systems [3, 4].

Tretinoin or all-trans retinoic acid is used topically as a cream, lotion or gel for the treatment of acne. The topical application of this molecule enhances collagen and hyaluronic acid production and is used as an anti-aging regimen. Oral administration of this drug is employed in the treatment of acute promyelocytic leukemia. However it has poor aqueous solubility, high chemical instability and it is prone to photodegradation, thermal degradation and oxidation. Tretinoin-loaded lipid-core nanocapsules have shown great enhancement in antitumoral activities as compared to the pure drug. Nanosuspensions enhance drug photostability, and chitosan solid-lipid nanoparticles improve topical delivery of tretinoin [5–9].

Tretinoin and erythromycin combination is effective in the treatment of acne. Tretinoin enhances erythromycin skin penetration and increases its efficiency and antibacterial activity by reducing cellular adhesion. This leads to drainage of excess sebum and creates an aerobic condition that may hinder the growth of anaerobic bacteria including *Propionibacterium acnes* [10, 11].

A combination of erythromycin and tretinoin has demonstrated enhanced efficacy in in vivo models. Thus the main purpose of this study was to develop tretinoin-loaded nanofibers as a potential facial anti-acne patch and investigate its physicochemical characteristics including drug release, and stability. We also aimed to investigate the possibility of synergism in the antibacterial activity of tretinoin and erythromycin combination on *Staphylococcus aureus (S. aureus)* strains including (ATCC® 25,923™) and (ATCC® 29,213™) by Kirby Bauer disc diffusion method.

## Materials and methods

### Materials

Tretinoin and Erythromycin were obtained from Sepidaj Pharmaceutical, Tehran, Iran. Polycaprolactone (PCL; Mw 70,000–90,000 by GPC) was purchased from Sigma-Aldrich. N, N-Dimethylformamide (DMF; Purity ≥99.0%) was obtained from Merck (Germany). *S. aureus* ATCC (American Type Culture Collection 25,923™) and *S. aureus* (ATCC® 29,213™) were purchased from Kwik-Stik Microbiologics, France. Mueller-Hinton Agar was obtained from Becton Dickinson, France.

### Electrospinning of nanofibers

A 10% w/v solution of polycaprolactone was prepared in dimethylformamide at 50 °C for 4 h. The drug was added to the polymeric solutions and placed in a syringe connected to a blunt 27 G stainless steel needle mounted on a syringe pump (syringe pump model SP1000HOM Fanavaran Nano-Meghyas, Iran) flowing at a constant rate of 1 ml/h. High voltage power supply (model HV35P OV; Fanavaran Nano-Meghyas, Iran) was applied at 19 kV and the distance between needle and collector was 16 cm. The electrospun nanofibers were collected on aluminum foils and dried under vacuum prior usage. Details of all formulations are shown in Table 1.

**Table 1:** Details of the nanofiber formulations and viscosity of electrospinning solutions

Formulation	Composition %w/v		Viscosity	
	PCL	Tretinoin	Viscosity	Torque
A	10	0.5	122.0	81.8%
B	10	1	123.1	82.4%
C	10	–	118.1	79.2%

### Solution viscosity and Nanofiber morphology

The viscosity of the polymeric solution was evaluated prior to electrospinning using a rotational viscometer (Evo expert viscometer; Fungilab, Spain) with a spindle L2 configuration at room temperature and a rotational speed of 200 rpm.

Fiber morphology was studied with field emission scanning electron microscopy (FESEM) (MIRA3; TESCAN, Czech Republic). Samples were sputter-coated with gold prior visualization in a high vacuum chamber at 20Kv. Nanofiber diameter was evaluated with Image Analysis software (*n* = 40).

### Fourier-transform infrared spectroscopy (FTIR) analysis

The functional groups of polymer and drug and the possible interactions between them were studied by FTIR (Spectrum Two; Perkin Elmer, USA) in the wavelength of 500–4000 cm^− 1^ at room temperature.

### Drug release

Drug release studies were performed using the total immersion method at 25 °C and 32 °C (this temperature was chosen to mimic the skin condition). A known amount of fiber was immersed in phosphate buffer saline, PBS (pH 7.4) placed on a shaker-incubator (GFL 3031, Germany). At specific time intervals, 1 ml samples were taken from the release medium and were replaced with the same amount of the fresh medium. Drug concentration was measured by spectrophotometer (CE7200; CECIL, United Kingdom) at the maximum wavelength of 352 nm.

### Storage stability

Tretinoin content in the nanofiber was quantified via spectrophotometer by the complete dissolution of a certain amount of fibers in a solvent. Loading dose (%) and entrapment efficiency were calculated using the following method [12]:

$$ \mathrm{Loading}\ \mathrm{dose}\ \left(\%\right)=\frac{\mathrm{Drug}\ \mathrm{content}\ \left(\mathrm{mg}\right)}{\mathrm{Nanofiber}\ \mathrm{weight}\ \left(\mathrm{mg}\right)}\times 100 $$

$$ \mathrm{Entrapment}\ \mathrm{efficiency}\ \left(\%\right)=\frac{\mathrm{Measured}\ \mathrm{drug}\ \mathrm{content}}{\mathrm{Theoretical}\ \mathrm{drug}\ \mathrm{content}}\times 100 $$

Nanofiber stability was investigated at room temperature (25 °C) and fridge (4–8 °C) for 45 days.

### Antimicrobial activity tests

Disc diffusion tests were performed using *S. aureus* ATCC (American Type Culture Collection 25,923™) and *S* (ATCC® 29,213™) after growth in sheep blood agar at 37 °C for 24 h. The produced colonies were inoculated as a single colony in Mueller-Hinton Agar containing Petri dishes (Ø 8 cm) overnight at 37 °C [13]. The punched electrospun nanofiber discs (6 mm) were incubated at 37 °C for 24 h on Muller Hinton Agar medium inoculated with bacteria suspension with turbidity adjusted to 0.5 McFarland standard. The inhibition zone was measured in millimeters (mm) using a ruler. Three replicate were carried out under the same conditions for each sample.

### Statistical analysis

All data are expressed as mean ± SD and statistical analysis was performed by GraphPad Prism using one way ANOVA and *p* < 0.05 was considered statistically significant (*n* = 3).

## Results

### Solution viscosity and Nanofiber morphology

**Fig. 1:**
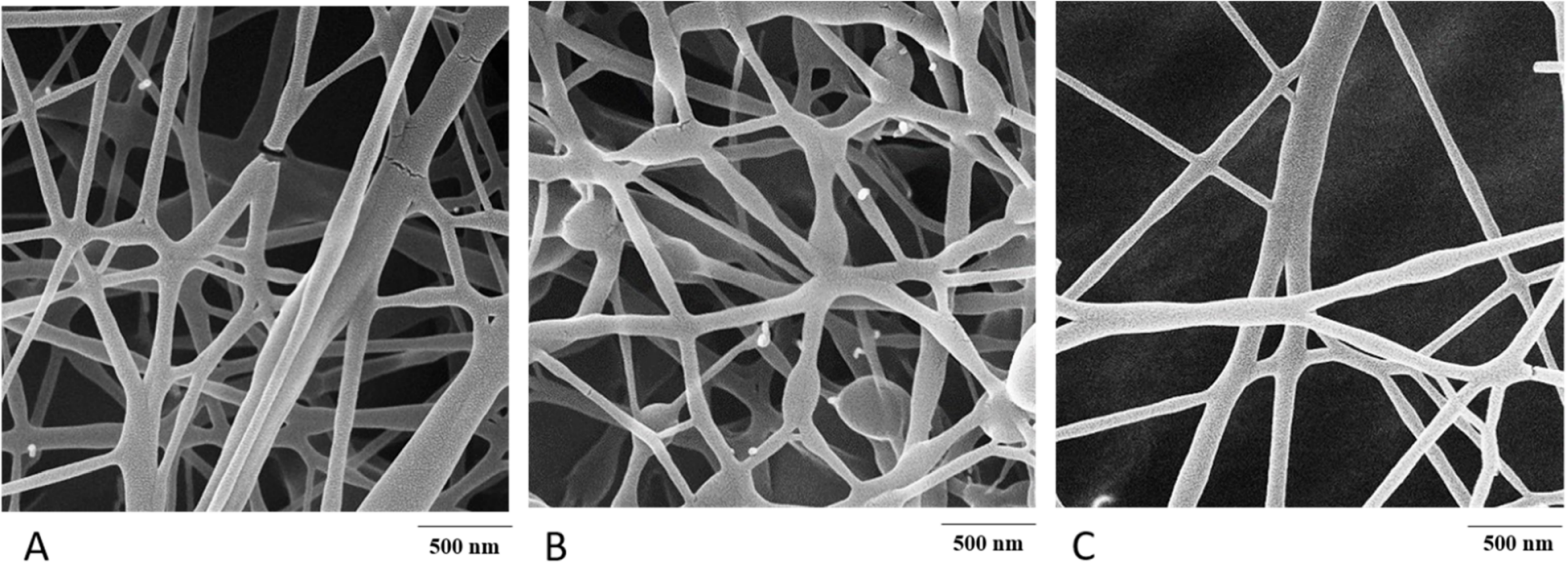
FESEM images of formulations **a**, **b** and **c**

The viscosity of electrospinning solutions was measured with a rotational viscometer (Table 1). It is seen that all formulations had similar viscosity. This may indicate that tretinoin, when used at such low concentrations did not influence solution viscosity. FESEM images of nanofiber formulations (Fig. 1) represent uniform fibers without drug crystals. The drug-loaded Fibers show gradual morphological changes with drug concentration. Formulations A and formulation C represent bead-free fibers with a diameter of 64.67 ± 15.78 nm and 80.7 ± 12.67 nm, respectively. Formulation B resulted in a mean fiber diameter of 66.81 ± 15.68 nm with some beads.

### FTIR analysis

**Fig. 2:**
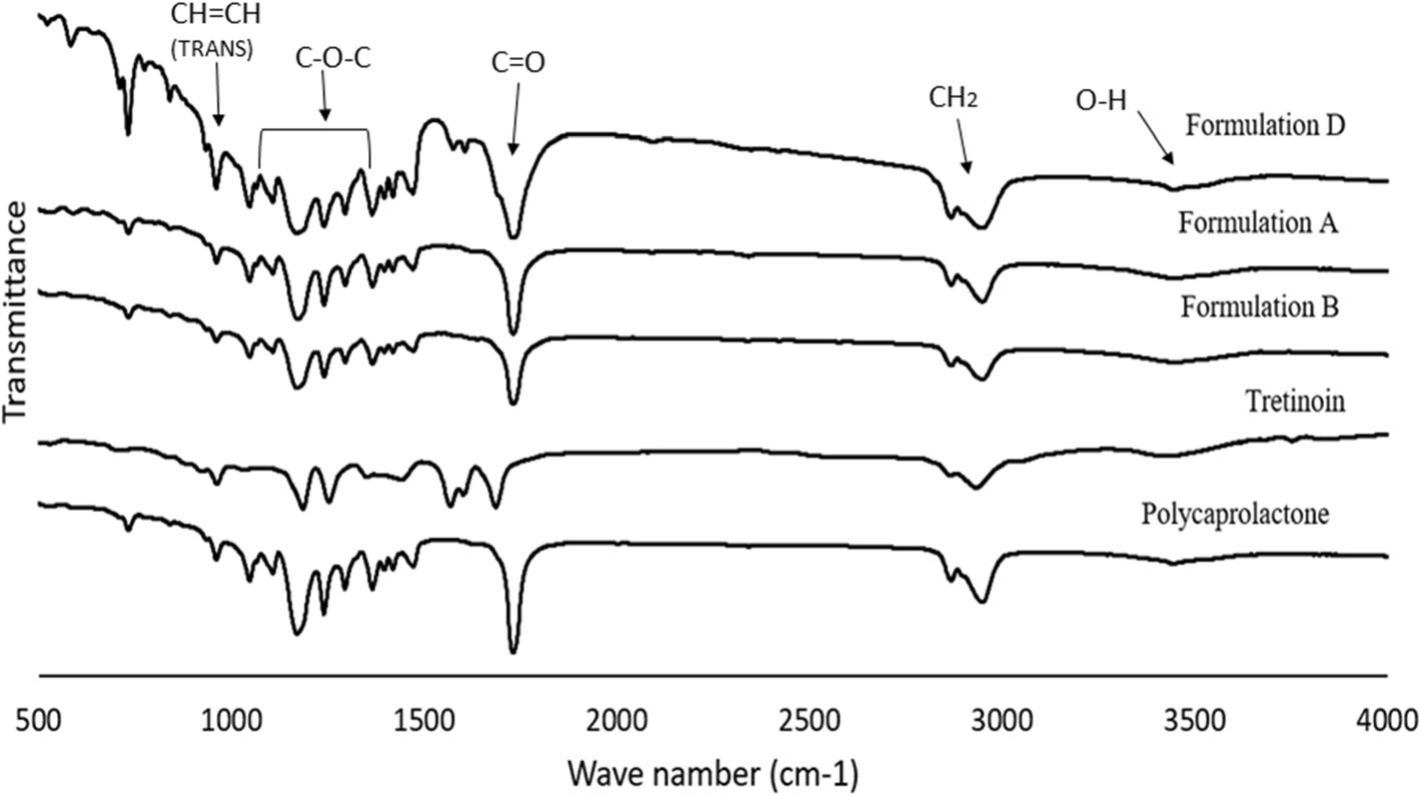
FTIR spectra of various formulations

The interaction between polymer and tretinoin and their functional groups were studied via FTIR (Fig. 2). In pure PCL peaks at 2864 cm^− 1^ and 2942 cm^− 1^ represent stretching vibrations of CH_2_, the peak in 1722 cm^− 1^ attributes to stretching vibrations of C=O and band in 1000–1300 cm^− 1^ indicates stretching vibrations of the C-O-C group of the PCL backbone. The peak in 3438 cm^− 1^ depicts O-H stretching vibrations, the band in 2324 cm^− 1^ corresponds to C=O stretching and peaks at 960 cm^− 1^ shows the trans vinyl (CH=CH) groups of tretinoin molecule [14–19].

### Drug release

**Fig. 3:**
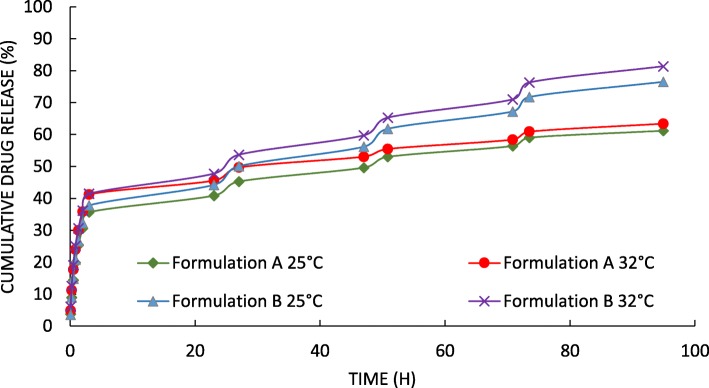
Cumulative drug release profile of formulation A (Tretinoin 0.5%w/v) and formulation B (1% w/v) at 25 °C and 32 °C

The tretinoin loading dose for formulation A and B was 4.76 and 7.94% respectively. Entrapment efficiency was found to be 99% for formulation A and 89% for formulation B. The in-vitro drug release profile was investigated over 90 h in phosphate buffer solution at 32 °C and 25 °C (Fig. 3). In both formulations, drug release was higher at 32 °C when compared to that of 25 °C (*P* < 0.05). Formulation A and B released 35.65 and 37.80% of their drug content in the first 3 h at 25 °C followed by a gradual drug release over the next 4 days. At 32 °C, 41.21 and 41.45% of drug content were released from formulation A and B respectively in the first 3 h.

### Storage stability

**Fig. 4:**
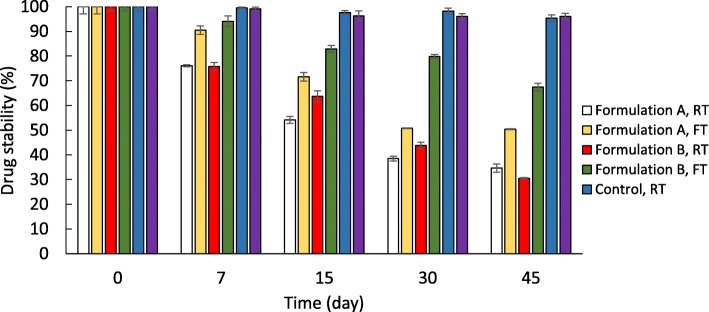
Storage stability of tretinoin in (formulations A (Tretinoin 0.5% w/v) and B (Tretinoin 1% w/v). Significance difference between nanofibers stability is observed while storage at room (RT) and fridge temperature (FT). * *P* < 0.05, ** *P* < 0.01, *** *P* < 0.001 for formulation A and # *P* < 0.05, ## *P* < 0.01, ### *P* < 0.001 for formulation B

Tretinoin storage stability was studied at room temperature (RT) and fridge temperature (FT) for 45 days and the results are shown in Fig. 4. At the end of 45 days, the drug content in formulations A and B was 34.7 and 30.5% respectively while storage at RT and 50.4 and 67.4% respectively at FT. Both formulations were significantly more stable in FT compared to RT after 45 days of storage (*P* < 0.05 for formulation A and *P* < 0.001 for formulation B).

### Antimicrobial activity tests

**Fig. 5:**
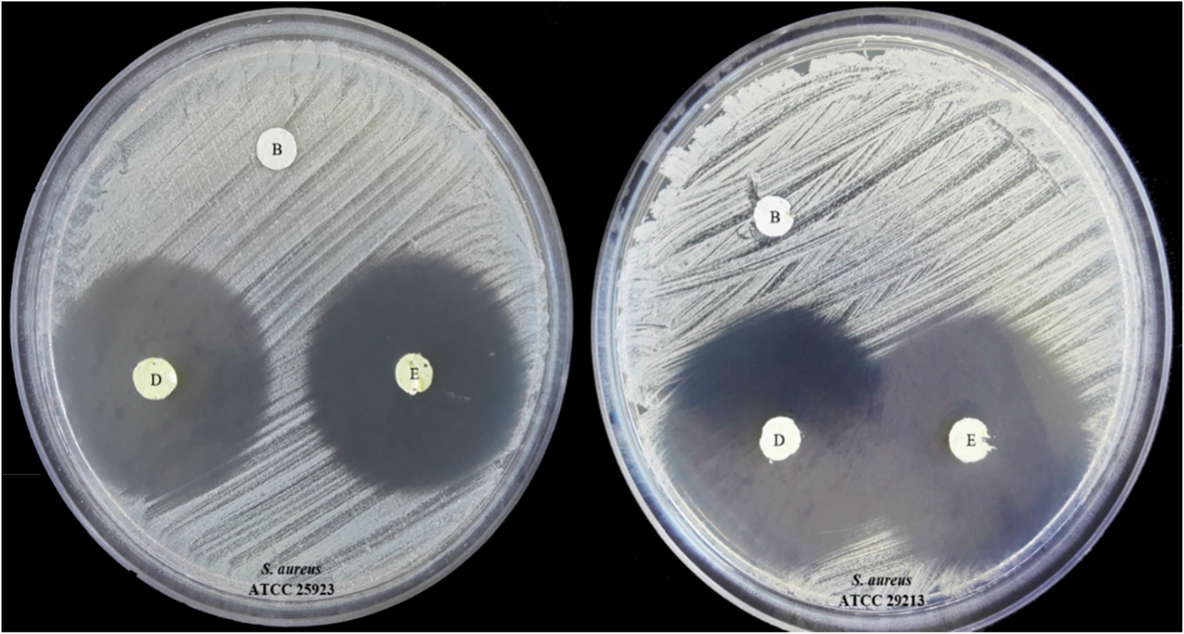
In-vitro antibacterial activity of formulations B (tretinoin 1% w/v), D (Tretinoin 1% w/v and Erythromycin 0.7%w/v) and E (Erythromycin 0.7% w/v) against *S. aureus* (ATCC® 25,923™) and *S. aureus* (ATCC® 29,213™). For formulation D and E there is no significant difference in the zone of inhibition

The possible synergism in the antibacterial activity of tretinoin and erythromycin combination was measured against *S. aureus* (ATCC® 25,923™) and (ATCC® 29,213™) by the disc diffusion method and the results are presented in Fig. 5 and Table 2. Results indicate that formulation A, B, and C did not display any antibacterial activity. Formulations D and E exhibited antibacterial activity against *S. aureus* (ATCC® 25,923™) with an inhibition zones of 31 mm. Antibacterial activity against *S. aureus* (ATCC® 29,213™) was similar for both formulation D and E and the inhibition zones were 32 mm. Nanofibers showed higher inhibition diameters compared to standard erythromycin disc. This enhancement in the antibacterial effect may be attributed to the higher surface to volume ratio of nanofibers. Tretinoin did not show synergistic antibacterial effects with erythromycin.

**Table 2:** Disc diffusion test and inhibition zone diameter (mm) of electrospun nanofibers and standard antibiotic discs against *S. aureus*

Strains	Sensitivity diameter (mm)						
	Formulation^a^					Standard antibiotic disc	
	A	B	C	D	E	^a^EM	^a^CTT
						15	30
*S.Aureus* ATCC®25,923	0	0	0	31	31	30	22
*S.Aureus* ATCC®29,213	0	0	0	32	32	30	23

^a^A: Tretinoin 0.5% w/v, B: Tretinoin 1% w/v, C: plain nanofiber with no drug, D: Tretinoin and Erythromycin-loaded nanofiber, E: Erythromycin-loaded nanofiber, and standard antibiotic disc including *EM* Erythromycin and *CTT* Cefotetan

## Discussion

Tretinoin-loaded nanofibers were successfully fabricated. It was found that formulations with a higher concentration of tretinoin had some bead formation. This may be due to the higher concentration of tretinoin which may increase some of the physicochemical properties of the solution including surface tension and hydrogenic interactions between tretinoin and polymer. Due to the complete incorporation of the drug in nanofiber beds, all formulations displayed crystal-free structures [14, 15]. FTIR results indicate that all characteristic peaks are present which signify the presence of drug molecule within the polymer backbone.

Drug release from the nanofibers was carried out at two different temperatures for a period of 4 days. Both formulations showed a similar biphasic release profile and a burst release in the first 3 h. This demonstrates the dissolution of the drug-loaded on the surface of nanofibers followed by a slow-release phase that may be due to diffusion of drug trapped inside the fibers [20]. Due to the semi-crystalline structure of PCL and its low degradation rate, part of the drug was trapped inside nanofibers which release with polymer degradation over time [21].

The chemical stability of tretinoin was tested in the developed formulations for 45 days at two different temperatures. It was found that the drug was significantly more stable in the fridge. Overall tretinoin is a vulnerable drug and is prone to instability. Results from previous studies exhibited low stability of tretinoin loaded polymeric micelles. The oxidation profile of this drug was investigated at various temperatures and it was found that tretinoin can easily oxidize even at low temperatures. Therefore decomposition by light and air should be prevented and the drug should be stored carefully to prevent oxidation. A significant decrease in drug degradation may occur when stored under an argon atmosphere, therefore a reasonable attempt should be made to decrease the degradation rate and protect from oxygen. Application of a stabilizer is another approach that can be used to enhance drug stability [5, 22].

Previous studies have demonstrated antibacterial activity for retinaldehyde. This effect may be due to the presence of the aldehyde group which may demonstrate bifunctional property for some retinoids. Results of the disc diffusion studies on two *S. aureus* strains in this work did not exhibit any synergistic antibacterial activity in erythromycin and tretinoin combination. These results are supported by previous findings were retinoic acid and retinol did not have any antibacterial effect against gram-positive and gram-negative bacteria. Similar in vitro results were reported for tretinoin-loaded solid lipid nanoparticles with no significant antimicrobial activity against *P. acnes* (ATCC 6919) and *S. aureus* (ATCC 29213) [9, 23].

Various in vivo studies however have demonstrated that tretinoin enhances antibiotics activity of erythromycin by inhibition of trans-glutaminase activity which reduces cellular adhesion and follicular plugging and leads to drainage of excess sebum and bacteria. Tretinoin may also help create a more aerobic condition which hinders the growth of anaerobic bacteria like *P. acnes.* Application of tretinoin and clindamycin phosphate significantly improved the antibacterial and anti-inflammatory effect at various stages of acne*.* Recently, a combination of a topical antibiotic and tretinoin are recommended for the treatment of acne [11, 24, 25].

The results of this work demonstrate that fiber formulation can offer a great advantage for the stability of tretinoin and provide sustained release of drug at the site of action. Preliminary results obtained in this work did not indicate any synergism in tretinoin and erythromycin combination in in vitro tests; however, this formulation may help enhance its therapeutic effect in vivo.

## Conclusion

The overall purpose of the study was to fabricate nanofibers containing tretinoin as a drug delivery system for the treatment of acne. Uniform fibers without drug crystals were fabricated via electrospinning. Formulations were investigated for drug release, stability and antimicrobial activity in combination with erythromycin. Electrospun nanofibers showed a prolonged release of tretinoin. The stability of formulations in FT was higher than RT. It can be anticipated that the easy fabrication, low costs and dosing frequency of the construct reported here provide a platform that can be adapted for on-demand delivery of tretinoin, either solo or in combination with erythromycin. Formulation changes and the addition of other pharmaceutical excipients for improving drug stability and release will be explored in future research.

## Data Availability

Data associated with this paper is available from the corresponding author upon reasonable request.

## Abbreviations

FESEM: Field emission scanning electron microscopy
FT: Fridge temperature
FTIR: Fourier-transform infrared spectroscopy
*P. acnes*: *Propionibacterium acnes*
PCL: Poly caprolactone
RT: Room temperature
*S. aureus*: *Staphylococcus aureus*
